# Abnormal Condition of the Digestive Organs

**Published:** 1878-01

**Authors:** Fred. King

**Affiliations:** Atlanta, Ga.


### ABNORMAL CONDITION OF THE DIGESTIVE ORGANS

By FRED. KING, M.D., Atlanta, Ga.

Notwithstanding the fact that a great deal has been written on dyspepsia, I think many physicians are almost entirely ignorant of its causes, and the many pathological conditions that are to be observed in the various forms of the disease. This is owing, in a great measure, to the fact that a majority of us do not regard indigestion of sufficient importance to merit a place among the serious maladies with which we have to deal. When consulted by dyspeptics we are too prone to regard them as hypochondriacs and therefore dismiss them after having prescribed a few bitter tonics and given some direction as to diet, etc. By such hasty and unjustifiable proceedings we do ourselves injury and our unfortunate patient injustice. If any relief is afforded, it is only temporary, and the sufferer finally seeks "quack" and "stomach bitter men," who are ever ready to fleece them of their last farthing.

All abnormal conditions of the digestive organs may be embraced under the one head, dyspepsia; and this term covers such a broad field that it is no easy task for us, when called upon to treat a patient suffering from indigestion, to reach a satisfactory diagnosis without thoroughly studying each individual case. We rarely meet two consecutive cases in which the same symptoms are present. This is owing, of course, to the fact that different organs connected with the functions of digestion are at fault. The symptoms, however, in all cases, are local; that is, they proceed directly from the stomach or small intestines—the first stage of digestion taking place in the former, the last in the latter.

When the symptoms are traceable to the stomach, we may often attribute them to a deficiency in the quantity or the quality of the gastric fluid. It is utterly impossible to enumerate, in a short paper like this, the various causes of this deficiency. We should always carry our investigations far enough to be able to locate, if possible, the exact seat of the trouble with which we have to deal, and then govern ourselves according to the result of our inquiries.

Under two principal or leading heads may be classified all forms of dyspepsia: *First*, labored or difficult digestion; and, *Second*, symptoms of disturbed or imperfect digestion. A sense of discomfort is always the result of indigestion of any kind, while, on the other hand, ease and perfect comfort always follow the proper performance of these important functions.

Dyspeptics are nearly always uneasy, or miserable, while the process of digestion is going on; in many instances this state amounts to the most extreme melancholy. These are oftentimes the only symptoms present. When such a condition as this exists, we may at once conclude that we are to deal with labored or difficult digestion, resulting from a deficiency of some of the elements of the gastric juice, and must therefore prescribe such remedies as are calculated to make up for the deficiency.

If the symptoms arise from disturbed or imperfect digestion, they are traceable to a point lower down in the alimentary canal.

The most prominent symptoms of indigestion—such as regurgitations, cardialgia, catharsis, tympanitis, vomiting, etc.—are so well known to us all, that I deem anything like an extended reference to them altogether superfluous. They all result from different causes—some originating in the stomach, while others come from a point below the pyloric orifice of that organ. An extended review of the many pathological changes taking place in these abnormal conditions of the organs engaged in digestion would be pertinent in this connection, did time and space permit, and would be very conducive to a better understanding of the subject under consideration. But I must hasten on to the consideration of the remedies which, in my opinion, are calculated to relieve many forms of dyspepsia.

In many cases of indigestion I am sure that the prime cause of the trouble may be found either in the stomach or pancreas, and I am satisfied that the latter organ is as often at fault as the former (hence the worthlessness of pepsin alone, in many cases). The symptoms, in a large majority of the cases that have come under my immediate observation, point strongly to the establishment of this proposition. I am decidedly inclined to the opinion that many cases of supposed "chronic diarrhœa" are nothing but indigestion dependent upon a want of proper emulcification. The fats and oily substances passing through the intestines without being duly emulcified, I think act as a mechanical cathartic, just as melted lard purges when swallowed in very large quantities. A careful examination of the fæces in each individual case would throw additional light on the diagnosis when diarrhœa is a leading symptom. The presence of undigested oily substances could be easily detected by solution in water.

Now, as we are taught by physiologists that a certain amount of organic matter (pepsin and pancreatine), lactic and hydrochloric acids, chloride of sodium, potash, lime and ammonia, are indispensable in the processes of digestion, assimilation and nutrition, does it not necessarily follow that a diminution in the quantity or quality of any of these would be followed by general impairment of the great functions of digestion? Should we not, therefore, employ such agents or remedies as would make up such deficiencies? In my experience, nothing has met so successfully all these indications and symptoms and given such universal satisfaction as pepsin, pancreatine and lactic acid, with the addition of muriatic acid in some form. My employment of these substances in the various forms of indigestion during last year, was suggested by chemical observation in regard to the power of these agents over the digestive process.

My experiments were made upon meats of various kinds, breadstuffs, etc., or about what usually constitutes an ordinary meal for an adult. I tried them, also, on coagulated albumen, and found their powers superior to that of anything else I had experimented with. One of the most important among the many advantages possessed by these remedies over all others suggested for the relief of indigestion is, that they contain the natural acids secreted by the stomach—lactic and hydrochloric—without which neither fatty substances nor coagulated albumen is changed by pepsin or pancreatine. Pepsin will not emulsify fats, nor will pancreatine dissolve coagulated albumen, but all of the substances mentioned will accomplish both.

And now, before reporting, or, rather, furnishing synopses of a few cases where they were prescribed, I will give my reasons for diminishing the dose from time to time. I did so from the fact that a certain amount of the elements contained in the remedies was absorbed, and of course found their way, through the medium of the circulation, back to the stomach, pancreas, etc., and again aided in the work they were first intended to assist in.

CASE 1.—Mr. C. consulted me early in September last, suffering from dyspepsia of long standing. The symptoms indicated that the trouble was near the pyloric orifice of stomach. I gave him the foilowing:

℞.—Lactopeptin, . . . . gr.clxxx.
Vin. Alb., . . . . f℥viij.

M. S.—Tablespoonful immediately after eating, three times a day, gradually reducing the dose to teaspoonful.

At the expiration of one month he expressed himself entirely cured.

CASE 2.—Mr. S. applied to me in August. He had been troubled with a serious diarrhœa for more than a month. After a careful diagnosis, I decided that indigestion was the prime cause of his trouble. The lactopeptin, administered in same way as in Case 1, relieved him entirely within a fortnight. (Mr. F. was prescribed for in same way on same day and the result was equally satisfactory.)

CASE 3.—Tinxy R., colored, married, aged 23; was enæmic, pregnant; suffered from cardialgia and vomiting. I gave her:

℞.—Elx. lactopeptin with bark and iron, . ℥vi.

SIG.—Dessert spoonful ter die.

The vomiting as well as all other bad symptoms soon disappeared.

CASE 4.—A little daughter of Dr. C. had capricious appetite; was restless. Various worm medicines had been employed without success. Upon my suggestion, the doctor gave her:

℞.—Syr. lactopeptin comp., . . . ℥ij.

S.—Teaspoonful, gradually diminishing to ten drops, three times daily after meals.

She commenced improving at once, and was soon restored to perfect health.

CASE 5.—Dr. B., a professional friend, had suffered for more than two years from neuralgia, which he attributed to indigestion. Ten grains of lactopeptin after each meal soon relieved.

Indeed, I find lactopeptin, or other forms of using pepsin, pancreatine and hydrochloric acid, superior to anything I have ever used in all forms of dyspepsia, intestinal diseases of children, chronic diarrhœa, constipation, vomiting in pregnancy, and in fact, in all troubles arising from abnormal conditions of the digestive organs. Different patients, however, require different forms of preparation. While adult males take the dry powder, liquid or wine, females or children require the elixir or syrup.

These remedies may be given in connection with ferruginous and bitter tonics, cod liver oil, etc., when desirable.

